# Development of a self-assessment tool for resident doctors’ communication skills in India

**DOI:** 10.3352/jeehp.2019.16.17

**Published:** 2019-06-24

**Authors:** Upendra Baitha, Piyush Ranjan, Siddharth Sarkar, Charu Arora, Archana Kumari, Sada Nand Dwivedi, Asmita Patil, Nayer Jamshed

**Affiliations:** 1 Department of Medicine, All India Institute of Medical Sciences, New Delhi, India; 2 Department of Psychiatry and National Drug Dependence Treatment Centre, All India Institute of Medical Sciences, New Delhi, India; 3 Department of Home Science, University of Delhi, New Delhi, India; 4 Department of Obstetrics and Gynaecology, VMMC and Safdarjung Hospital, New Delhi, India; 5 Department of Biostatistics, All India Institute of Medical Sciences, New Delhi, India; 6 Department of Physiology, All India Institute of Medical Sciences, New Delhi, India; 7 Department of Emergency Medicine, All India Institute of Medical Sciences, New Delhi, India; Hallym University, Korea

**Keywords:** India, Patient care, Physician-patient relations, Self-assessment

## Abstract

**Purpose:**

Effective communication skills are essential for resident doctors to provide optimum patient care. This study was conducted to develop and validate a questionnaire for the self-assessment of resident doctors’ communication skills in India.

**Methods:**

This was a mixed-methods study conducted in 2 phases. The first phase consisted of questionnaire development, including the identification of relevant literature, focus group discussions with residents and experts from clinical specialties, and pre-testing of the questionnaire. The second phase involved administering the questionnaire survey to 95 residents from the Departments of Medicine, Emergency Medicine, Pediatrics, and Surgery at the All India Institute of Medical Sciences, New Delhi, India in April 2019. Internal consistency was tested and the factor structure was analyzed to test construct validity.

**Results:**

The questionnaire consisted of 3 sections: (A) 4 items on doctor-patient conflicts and the role of communication skills in avoiding these conflicts, (B) 29 items on self-assessment of communication skills in different settings, and (C) 8 items on barriers to practicing good communication skills. Sections B and C had good internal consistency (Cronbach α: 0.885 and 0.771, respectively). Section C had a 2-factor solution, and the barriers were classified as ‘training’ and ‘infrastructure’ factors.

**Conclusion:**

This appears to be a valid assessment tool of resident doctors’ communication skills, with potential utility for identifying gaps in communication skills and developing communication skills modules.

## Introduction

Effective doctor-patient communication is a prerequisite for a successful and robust healthcare delivery system. It not only helps the doctor to earn the patient’s trust and confidence, but also improves patients’ overall satisfaction and compliance with treatment [1]. In developing countries such as India, healthcare delivery systems are constrained in terms of resources and societal expectations of healthcare providers. Doctors are often overwhelmed by the number of patients and their family members that have to be catered to. Ineffective referral systems and asymmetric incentives and regulations also lead to patients and their caregivers sometimes being disgruntled with healthcare services. This may result in emotional outbursts from the patients and their attendants, in some cases leading to violence towards doctors and healthcare staff [2,3]. In teaching hospitals, residents are often the first point of contact for the patients and their care providers, and they often face the brunt of such expressions of discontentment. Studies suggest that one of the main reasons for doctor-patient conflicts is the lack of proper communication between both parties [4]. Hence, in recent decades, substantial importance has been placed on improving communication skills training in the medical curriculum [5,6].

Regular assessments are an important component of any training program. Multiple assessment methods such as evaluations by peer groups, patients, and nurses have been studied worldwide to ascertain communication skills in doctors. These assessment tools use checklists, rating scales, and subjective opinions to rate these skills. Although valid and reliable, these tools are resource-intensive and complex to use and interpret. Furthermore, these instruments seldom capture some of the specific issues and challenges in communication that are faced in non-Western systems. We undertook this study to develop and validate a simple and generic self-assessment questionnaire that could be relevant for most clinical specialties in assessing the communication skills of resident doctors. Furthermore, we sought to develop a questionnaire that could be used to estimate the extent of doctor-patient conflict, and to ascertain the barriers to good communication by resident doctors.

## Methods

### Ethics statement

This study was conducted after receiving approval from the Institutional Ethics Committee (IEC/174/3/2018) and informed consent was obtained from all participants.

### Study design

A mixed-methods study for questionnaire development and validation.

### Phase 1: Questionnaire development

A systematic methodology was used in the development of this questionnaire, including the following 4 main steps: literature review, focus group discussions (FGDs), expert evaluation, and pre-testing [7].

The first step, literature review, included electronic searches of Google Scholar and PubMed. The keywords included in the searches were “barrier in communication,” “communication skills,” “doctor-patient conflict,” “evaluation,” “interns,” “medical students,” and “resident doctors.” After screening titles, abstracts, and full texts, relevant papers were selected and were read in-depth to identify relevant items. The initial search resulted in a short-list of 206 related articles, 78 of which were found to be relevant. From those 78 articles, 30 items were generated.

The second step involved FGDs and item generation. Three FGDs were conducted: 2 with residents and a subsequent FGD with faculty experts. The 2 FGDs with residents included 10 participants from 5 different clinical specialties (emergency medicine, surgery, medicine, pediatrics, and infectious diseases). As a result of a detailed literature review and FGDs, a list of items was generated that adequately represented the construct of the questionnaire. This list contained 45 items (30 from the literature search and 15 additional locally relevant items identified from FGDs). Attention was given to proper sequencing and framing of questions in simple language. Each of these items was carefully designed to make reference to a single concept, to be positively worded, to avoid double negatives and ambiguity, and to use expressions in the first person. The items covered a comprehensive range of communication skills for residents, including verbal, non-verbal, and para-verbal communication; breaking bad news; understanding team dynamics; understanding patients’ perspectives and expectations; sharing information with patients; involving patients in decision-making; and identifying barriers to good communication skills.

The generated items were discussed with faculty members from several clinical departments in an FGD. Opinions on the items were also solicited from 12 experts with expertise in the fields of communication skills, psychiatry and clinical psychology, biostatistics, and pediatrics. In accordance with their suggestions, 3 items were re-worded and 3 were deleted. The subsequent draft version of the questionnaire was pre-tested by 20 residents to check for comprehensibility, acceptance and ease of usage of the designed tool. A 5-point Likert scale was employed for the response options, assuming an equal distance between response objects. Further refinement of the questionnaire was done at this stage to incorporate inputs from residents after pre-testing.

### Phase 2: Validation study

In this phase, the questionnaire was administered to 100 residents from the Departments of Medicine, Emergency Medicine, Pediatrics, and Surgery at the All India Institute of Medical Sciences, New Delhi, India and 95 completed responses were analyzed. The questionnaires were anonymized and were completed by the residents at their convenience. Data collection was conducted in April 2019. Descriptive statistics were used for analyzing demographic and clinical parameters. For the quantitative parameters, mean, median, standard deviation, quartile, and range were calculated. The Cronbach α was used to assess internal consistency (i.e., the extent to which the items on the instrument measure the same thing). Cronbach α values of 0.7 or higher are considered to indicate good internal consistency. Exploratory factor analysis was performed to examine the subdomain substructure. This technique is used to estimate factors and/or to reduce the dimensionality of a large number of variables to a fewer number of factors. The Kaiser-Mayer-Olkin (KMO) measure is used to assess sample adequacy, and values of more than 0.5 show that the data are suitable for factor analysis. The Bartlett’s test of sphericity is a statistical test for the overall significance of all correlations within a correlation matrix. Eigenvalues represent the variance in the variables that is accounted for by a specific factor.

## Results

The final questionnaire, approved by experts and pilot-tested on residents for its content and face validity, has 3 parts and is freely available for use. Section A comprises of general information and demographic data, and also contains 4 more questions (A1 to A4) that focus on doctor-patient conflicts and the role of communication skills in avoiding these conflicts. Section B is composed of 29 items (B1 to B29) and emphasizes the self-assessment of communication skills in different settings. This section is further divided into 4 domains, covering important aspects such as components of communication, dealing with patients in outpatient and intensive-care settings, breaking bad news, and communication with colleagues. Section C contains 8 questions (C1 to C8) and pertains to barriers to practicing good communication skills. The questionnaire takes roughly 20 to 25 minutes to complete. The final questionnaire is available in Supplement 1. The raw data are available in Supplement 3.

### Demographic findings of study subjects

The demographic details of the 95 included residents are presented in Table 1. About three-fourths of the participants were male and a similar proportion belonged to the Department of Emergency Medicine. The mean age of the residents was around 28 years, with an average of approximately 3 years of clinical experience.

### Descriptive statistics of survey results

The responses to sections A, B, and C of the questionnaire are presented in Supplement 2. It was found that nearly 75% of participants had never faced an episode of physical violence with a patient or attendant (caregivers, family members, and/or community members who accompany a patient) at their workplace; however, 33% of residents had experienced minor conflicts with patients or attendants at least once a week, and 8% of residents reported the daily occurrence of major doctor-patient conflicts. Almost 45% of residents were of the opinion that three-fourths of doctor-patient conflicts can be prevented by good communication practices. In section B, pertaining to the self-assessment of communication skills, displaying appropriate courtesy while communicating with nurses, paramedical staff, and other support staff was most commonly endorsed, while answering queries when attendants gather information from the internet or other sources was least frequently endorsed. In section C, lack of time, infrastructure deficits, and long working hours were reported as the major barriers to practicing good communication skills, by almost 50% of the residents.

### Construct validity of the survey tool

Factor analysis via principal component analysis using varimax rotation was run on each of the sections of the questionnaire. An eigenvalue of 1 was used as a cut-off for determining the number of factors, though the scree plot also gave an estimate for the number of tenable factors. For section A, a single-factor solution was found to be most suitable and explained 47.0% of the variance (KMO=0.576, Bartlett’s test of sphericity P-value <0.001). For section B, an eigenvalue of 1 gave a 10-factor solution that explained 71.4% of the variance (KMO=0.721, Bartlett’s test of sphericity P-value <0.001). Items 3, 11, 21, and 23 from section B did not have a factor loading of more than 0.5 on any of the factors (i.e., they did not load on any of the factors) and were therefore removed. Section C of the questionnaire had a 2-factor solution that explained 61.3% of the variance (KMO=0.735, Bartlett’s test of sphericity P-value <0.001). Items 1, 3, 7, and 8 loaded on the first factor (labeled as ‘training’), and items 2, 4, 5 and 6 loaded on the second factor (labeled as ‘infrastructure’).

### Reliability of the survey tool

The questionnaire showed fair overall internal consistency (Cronbach α: 0.885 and 0.771 for sections B and C, respectively) and the Cronbach α values for all sections of the questionnaire are reported in Table 2.

## Discussion

We developed and validated this questionnaire-based tool by using a standard process that has recently been applied to develop some other questionnaires [8,9]. The questionnaire consists of items dealing with the self-assessment of communication skills in resident doctors, identification of barriers to practicing good communication skills, and estimation of the burden of doctor-patient conflicts. There are many possible barriers that affect a doctor’s communication skills. Inadequate knowledge and training in communication skills, language barriers, and human failings such as fatigue, stress, and lack of time are some common barriers that affect doctor-patient communication.

This questionnaire is a concise, comprehensive, easy-to-administer, and user-friendly tool, which will enable an easy and quick assessment of important components of communication skills among resident doctors from different clinical departments. The significant role of effective doctor-patient communication in the healthcare system has motivated many researchers worldwide to develop tools to assess communication-related competence among doctors. Such assessment methods must be reliable, valid, and specific. Surveys (qualitative or quantitative) and recordings (audio, video, or standardized observations) are the 2 broad categories of tools used to assess doctor-patient communication. Questionnaire-based surveys are easy to administer and are fairly accurate. The MAAS-Global is a widely used, valid, and reliable instrument for assessing doctor-patient communication skills. The 2-dimensional structure of the instrument makes it valuable to optimize teaching methods in communication skills training [10]. The Wayne State University School of Medicine, Detroit, Michigan uses resident self-ratings with the Kalamazoo Essential Elements Communication Checklist–Adapted as part of the objective structured clinical examination to promote resident self-reflection [11]. The questionnaire for self-assessment of communication skills developed in this study is also based on self-assessment and requires self-reflection and self-monitoring, which are essential for the process of lifelong learning and improvement. As compared to instruments eliciting specialty-specific communication-based competencies [12], self-assessment of communication skills has the potential to be applicable across various clinical specialties.

A unique feature of the questionnaire for self-assessment of communication skills developed in this study is that it assesses communication barriers along with communication skills. The barriers include infrastructural barriers such as lack of space, which are common in the developing world, but are unlikely to be experienced in resource-replete settings. The tool also gathers information about experiences of conflicts and violence, which are realistic occurrences and are intertwined with communication difficulties and breakdown. The psychometric properties of the tool vary across the 3 sections, as they are conceptually diverse. While section A covers experiences of conflicts and physical violence, it also has a question soliciting respondents’ opinion on whether conflicts can be avoided with good communication. Factor analysis revealed a single factor, showing a unidimensional construct, though the disparate nature of the items may account for the low internal consistency of this section. Section B, on the self-assessment of communication skills, had good internal consistency, suggesting that the questions functioned together as a whole. A good factor analytic solution could not be found, implying that a reductionist approach might not be robustly applicable to classify these items into groups. Section C, on barriers to communication, had good internal consistency and a clear factor structure with intuitive and heuristic implications for classifying barriers into those that apply to residents (training) and those that apply to the system (infrastructure).

The scope of useful applications of this questionnaire could be manifold. Firstly, it could be used periodically to encourage residents to reflect upon their communication skills and identify areas for improvement. Secondly, it can provide insights into the common issues faced by many residents and help design skills development programs for them. Thirdly, the questionnaire is expected to be useful for identifying systemic and curricular barriers to developing communication skills and for making changes in infrastructure and training to address these barriers. Fourthly, it can also be of use for the healthcare system, by helping to gauge the extent of conflicts and violence in the healthcare workplace and to identify measures to address those issues.

Some of the limitations of this study are the possibility of reporting bias, although efforts were made to reduce this by keeping the responses anonymous; the inclusion of only a handful of departments, with a skewed number of respondents from emergency medicine; the inability to establish predictive/concurrent validity, which would have required long-term follow-up; and the study being conducted at a single center. Additionally, limited aspects of communication were covered (although a systematic item development process was implemented), and there certain aspects may not have been covered in this study.

In conclusion, the questionnaire developed in this study provides a reliable and valid tool for the self-assessment of communication skills of resident doctors and provides them with feedback about their strengths and weaknesses. This self-assessment questionnaire has the potential to elicit behavioral changes in residents, potentially resulting in better patient outcomes. Responses from this questionnaire can also help to foster the awareness and focus required to improve medical communication training and assessment.

## Figures and Tables

**Table 1.** Demographic characteristics of the participants

Characteristic	Value
Age (yr)	28.12±3.19
Duration of clinical experience (mo)	35.04±28.81
Gender	
Male	71 (74.74)
Female	24 (25.26)
Specialty	
Medicine	15 (15.79)
Emergency medicine	66 (69.47)
Pediatrics	8 (8.42)
Surgery	6 (6.32)

Values are presented as mean±standard deviation or frequency (%).

**Table 2.** Internal consistency of the questionnaire

Section	Cronbach α	Variance (%)	KMO and Bartlett’s test of sphericity
A	0.497	47.0	KMO=0.576
			Bartlett’s test of sphericity: P-value <0.001
B	0.885	71.4	KMO=0.721
			Bartlett’s test of sphericity: P-value <0.001
C	0.771	61.3	KMO=0.735
			Bartlett’s test of sphericity: P-value <0.001

KMO, Kaiser-Meyer-Olkin measure.

## Supplementary materials

Supplement 1. Questionnaire for self-assessment of communication skills in resident doctors

Supplement 2. Frequency of residents’ responses to the validation survey

Supplement 3. Data files are available from https://doi.org/10.7910/DVN/E0YZ8G.

Supplement 4. Audio recording of the abstract.
