# Supplementary material for: Development of a self-assessment tool for resident doctors’ communication skills in India
Source: J Educ Eval Health Prof. 2019 Jun 24;16:17. doi: 10.3352/jeehp.2019.16.17 (PMC6635607; doi:10.3352/jeehp.2019.16.17)
Supplement: Supplementary file 1 — Supplement 1. Questionnaire for self-assessment of communication skills in resident doctors [file jeehp-16-17-suppl1.pdf]

Supplement 1. Questionnaire for self-assessment of communication skills in resident doctors

Age: ___________ Gender: _________________ Specialty: _____________________________________
Duration of clinical experience: Internship (12 months)+Junior Residency (________ months)+Senior Residency (___________)

Section A

A1. How often do you experience minor conflicts with a patient and/or attendants (caregivers/family members, people who accompany the patient) while fulfilling clinical responsibilities at your workplace?

[Minor conflict: an incident of unwarranted argument/debate resulting in *argumentative discussions, shouting, etc.* but not verbal abuse or physical violence]

a. Nearly daily b. About once a week
c. About once a month d. About once every 6 months
e. About once a year or less

A2. How often do you experience major verbal conflicts (exchange of verbal abuse) with patients and/or attendants while fulfilling clinical responsibilities at your workplace?

a. About once a week b. About once a month
c. About once every 3 months d. About once every 6 months
e. About once a year or less

A3. How many times have you encountered physical violence with patients and/or attendants while fulfilling clinical responsibilities at your workplace?

a. Four times or more b. Three times c. Two times d. Once e. None

A4. In your opinion, what proportion of doctor-patient conflicts can be avoided by good communication practice (i.e., patiently listening to your patients, fully explaining the nature and yield of investigations, benefits of treatment, course and prognosis of the disease)

a. Almost all b. About 75% c. About 50% d. About 25% e. None

Section B: Self-Assessment of Communication Skills

Effective communication has three basic components: verbal, non-verbal and paraverbal. The verbal component deals with the content of the message including the selection of words. The non-verbal component includes body language like posture, gesture, facial expression, and spatial distance. The paraverbal component includes tone, pitch, pacing, and volume of the voice. Questions 1–5 mostly address points pertaining to these aspects:

B1. While meeting a patient, I greet him/her warmly with a smile/do namaste/say hello

a. Always b. Often c. Some times d. Occasionally e. Rarely

B2. I prefer to address the patient by name during history taking/examination or interview

a. Always b. Often c. Some times d. Occasionally e. Rarely

B3. I make eye contact during conversation or interview

a. Always b. Often c. Some times d. Occasionally e. Rarely

B4. While a patient is talking, I try to avoid any interruption such as taking calls or checking messages
a. Always b. Often c. Some times d. Occasionally e. Rarely

B5. I pay attention to non-verbal cues like gestures and facial expressions of the patients
a. Always b. Often c. Some times d. Occasionally e. Rarely

Content and setting of discussion/interview sessions is an important aspect of communication with patients/attendants in the outpatient department/indoor setting. Questions 6–12 mostly address points pertaining to this aspect:

B6. I ensure privacy while conducting interview/discussion sessions with patients/attendants
a. Always b. Often c. Some times d. Occasionally e. Rarely

B7. I prefer simple language and avoid medical jargon and abbreviations
a. Always b. Often c. Some times d. Occasionally e. Rarely

B8. I explain the nature, course, and prognosis (both short term and long term) of the disease in detail
a. Always b. Often c. Some times d. Occasionally e. Rarely

B9. I explain in detail regarding the necessity and feasibility of expensive investigations and their effect on the course and outcome of the disease
a. Always b. Often c. Some times d. Occasionally e. Rarely

B10. I explain in detail regarding various treatment options available and their effect on the course and outcome of the disease
a. Always b. Often c. Some times d. Occasionally e. Rarely

B11. I involve the patient in the decision-making regarding the choice of investigation and treatment
a. Always b. Often c. Some times d. Occasionally e. Rarely

B12. Before concluding the interview, I ask the patient if he/she would like additional information
a. Always b. Often c. Some times d. Occasionally e. Rarely

Communicating with patients and attendants in indoor/high dependency unit/intensive care unit is challenging. Questions 13–18 are mostly related to the aspect of communicating with patients/attendants in this setting:

B13. When attendants gather information from the internet or other sources, I try to answer their queries by giving better references
a. Always b. Often c. Some times d. Occasionally e. Rarely

B14. While discussing the daily progress of the patients, I emphasise the dynamic nature of the disease
a. Always b. Often c. Some times d. Occasionally e. Rarely

B15. When a patient is seriously ill, I inform him/her about the course and prognosis of the disease multiple times during a day with the attendants
a. Always b. Often c. Some times d. Occasionally e. Rarely

B16. I talk to attendants and discuss in detail after visiting the patient's bed

a. Always b. Often c. Some times d. Occasionally e. Rarely

B17. I take consent from patients/attendants myself

a. Always b. Often c. Some times d. Occasionally e. Rarely

B18. I take consent from patients/attendants after detailed discussion

a. Always b. Often c. Some times d. Occasionally e. Rarely

Bad news means any information that has the potential to have a devastating influence on one's life. Breaking bad news is challenging for any doctor. Questions 19 to 23 deal with the different steps involved in the practice of breaking bad news.

B19. Before breaking bad news, I plan in advance and mentally rehearse the act of disclosure

a. Always b. Often c. Some times d. Occasionally e. Rarely

B20. Before breaking bad news, I tend to assess relative's/patient's knowledge and attitude by asking open-ended questions

a. Always b. Often c. Some times d. Occasionally e. Rarely

B21. While breaking bad news, I tend to give information in small portions rather than doing so abruptly

a. Always b. Often c. Some times d. Occasionally e. Rarely

B22. After breaking bad news, I address/attend to patient's emotional reaction with full patience

a. Always b. Often c. Some times d. Occasionally e. Rarely

B23. After breaking bad news, I discuss the future plan of treatment with the patients and/or attendants

a. Always b. Often c. Some times d. Occasionally e. Rarely

Team dynamics is important in the success of a treating team. The practice of good communication skills with colleagues, nurses, paramedical staffs and other supporting staff is extremely important. Questions 24 to 29 address this aspect:

B24. While communicating with nurses, paramedical staff, and other supporting staff, I display appropriate courtesy

a. Always b. Often c. Some times d. Occasionally e. Rarely

B25. While communicating with nurses, paramedical staff and other supporting staff, I highlight that their role and responsibility is equally important

a. Always b. Often c. Some times d. Occasionally e. Rarely

B26. I avoid criticising colleagues or having debates in front of patients or attendants

a. Always b. Often c. Some times d. Occasionally e. Rarely

B27. To motivate nurses, paramedical staff and other supporting staff; I teach them the principles of management of commonly encountered diseases in the ward

a. Always b. Often c. Some times d. Occasionally e. Rarely

B28. As a part of giving feedback, I regularly express appreciation for nurses, paramedical staff, and other supporting staff
a. Always b. Often c. Some times d. Occasionally e. Rarely

B29. As a part of giving feedback, I don't hesitate to give positive criticism/constructive suggestion to my subordinates and supporting healthcare staff
a. Always b. Often c. Some times d. Occasionally e. Rarely

Section C: Barriers to Practicing Good Communication Skills

In your opinion, to what extent do you agree/disagree that the following barriers prevent you from practicing good communication skills (i.e., listening to patients adequately, explaining in detail the nature and yield of investigations, course and prognosis of the disease, benefits of treatment, etc.)

C1. Lack of insight (I never felt that good communication is equally important in the management of diseases)
a. Strongly agree b. Agree c. Neutral d. Disagree e. Strongly disagree

C2. Lack of time
a. Strongly agree b. Agree c. Neutral d. Disagree e. Strongly disagree

C3. Difficulty in understanding the patient's language
a. Strongly agree b. Agree c. Neutral d. Disagree e. Strongly disagree

C4. Human failings like stress and fatigue
a. Strongly agree b. Agree c. Neutral d. Disagree e. Strongly disagree

C5. Infrastructural deficits like lack of proper place for discussion, overcrowding.
a. Strongly agree b. Agree c. Neutral d. Disagree e. Strongly disagree

C6. Long working hours
a. Strongly agree b. Agree c. Neutral d. Disagree e. Strongly disagree

C7. Lack of subject knowledge required for fully explaining the modalities of diagnosis, treatment options, or prognosis
a. Strongly agree b. Agree c. Neutral d. Disagree e. Strongly disagree

C8. Lack of training in communication skills
a. Strongly agree b. Agree c. Neutral d. Disagree e. Strongly disagree
