# Supplementary material for: Development of a self-assessment tool for resident doctors’ communication skills in India
Source: J Educ Eval Health Prof. 2019 Jun 24;16:17. doi: 10.3352/jeehp.2019.16.17 (PMC6635607; doi:10.3352/jeehp.2019.16.17)
Supplement: Supplementary file 2 — Supplement 2. Frequency of residents’ responses to the validation survey [file jeehp-16-17-suppl2.pdf]

Supplement 2. Frequency of residents' responses to the validation survey

Q. no.	Question	No. (%)
**Section A**		
A1	Frequency of experiencing minor conflicts with patients/attendants at workplace	
	Nearly daily	23 (24.21)
	Almost once a week	31 (32.63)
	About once a month	17 (17.89)
	About once in 6 months	16 (16.84)
	About once a year or less	8 (8.42)
A2	Frequency of experiencing major conflicts with patients/attendants at workplace	
	Nearly daily	8 (8.42)
	Almost once a week	17 (17.89)
	About once a month	11 (11.58)
	About once in 6 months	17 (17.89)
	About once a year or less	42 (44.21)
A3	Frequency of encountering physical violence with patients/attendants at workplace in lifetime	
	Four times or more	4 (4.21)
	Three times	0
	Two times	3 (3.16)
	Once	17 (17.89)
	None	71 (74.74)
A4	Proportion of conflicts that can be avoided with good communication practices	
	Almost all	15 (15.79)
	About 75%	44 (46.32)
	About 50%	27 (28.42)
	About 25%	9 (9.47)
	None	0
**Section B**		
Q. no.	Question	No. (%)
B1	While meeting a patient, I greet him/her warmly with a smile/do namaste/say hello	
	Always	11 (11.57)
	Often	50 (52.63)
	Sometimes	25 (26.31)
	Occasionally	6 (6.31)
	Rarely	3 (3.15)
B2	I prefer to address the patient by name during history taking/examination or interview	
	Always	41 (43.15)
	Often	26 (27.36)
	Sometimes	14 (14.73)
	Occasionally	7 (7.36)
	Rarely	7 (7.36)
B3	I make eye contact during conversation or interview	
	Always	62 (65.26)
	Often	28 (29.47)
	Sometimes	3 (3.15)
	Occasionally	2 (2.10)
B8	I explain the nature, course, and prognosis (both short-term and long-term) of the disease in detail	
	Always	39 (41.05)
	Often	43 (45.26)
	Sometimes	9 (9.47)
	Occasionally	3 (3.15)
	Rarely	1 (1.05)

(Continued to the next page)

Supplement 2. Continued

Section B		
Q. no.	Question	No. (%)
B9	I explain in detail regarding the necessity and feasibility of expensive investigations and their effect on the course and outcome of the disease	
	Always	45 (47.36)
	Often	37 (38.94)
	Sometimes	10 (10.52)
	Occasionally	3 (3.15)
	Rarely	0
B10	I explain in detail regarding various treatment options available and their effect on the course and outcome of the disease	
	Always	38 (40.00)
	Often	32 (33.68)
	Sometimes	20 (21.05)
	Occasionally	5 (5.26)
	Rarely	0
B11	I involve the patient in the decision-making regarding the choice of investigation and treatment	
	Always	24 (25.26)
	Often	39 (41.05)
	Sometimes	27 (28.42)
	Occasionally	4 (4.21)
	Rarely	1 (1.05)
B12	Before concluding the interview, I ask the patient if he/she would like additional information	
	Always	25 (26.31)
	Often	33 (34.73)
	Sometimes	21 (22.10)
	Occasionally	10 (10.52)
	Rarely	6 (6.31)
B13	When attendants gather information from the internet or other sources, I try to answer their queries by giving better references	
	Always	21 (22.10)
	Often	25 (26.31)
	Sometimes	31 (32.63)
	Occasionally	15 (15.78)
	Rarely	3 (3.15)
B14	While discussing the daily progress of the patients, I emphasise the dynamic nature of the disease	
	Always	29 (30.52)
	Often	35 (36.84)
	Sometimes	23 (24.21)
	Occasionally	8 (8.42)
	Rarely	0
B15	When a patient is seriously ill, I inform him/her about the course and prognosis of the disease multiple times during a day with the attendants	
	Always	40 (42.10)
	Often	34 (35.78)
	Sometimes	19 (20.00)
	Occasionally	2 (2.10)
	Rarely	0
B16	I talk to attendants and discuss in detail after visiting patient's bed	
	Always	33 (34.73)
	Often	33 (34.73)
	Sometimes	20 (21.05)
	Occasionally	8 (8.42)
	Rarely	1 (1.05)

(Continued to the next page)

Supplement 2. Continued

Section B		
Q. no.	Question	No. (%)
B17	I take consent from patients/attendants myself	
	Always	46 (48.42)
	Often	32 (33.68)
	Sometimes	11 (11.57)
	Occasionally	5 (5.26)
	Rarely	1 (1.05)
B18	I take consent from patients/attendants after detailed discussion	
	Always	46 (48.42)
	Often	31 (32.63)
	Sometimes	15 (15.78)
	Occasionally	12 (12.63)
	Rarely	1 (1.05)
B19	Before breaking bad news, I plan in advance and mentally rehearse the act of disclosure	
	Always	33 (34.73)
	Often	40 (42.10)
	Sometimes	11 (11.57)
	Occasionally	6 (6.31)
	Rarely	5 (5.26)
B20	Before breaking bad news, I tend to assess relatives'/patient's knowledge and attitude by asking open-ended questions	
	Always	29 (30.52)
	Often	40 (42.10)
	Sometimes	18 (18.94)
	Occasionally	5 (5.26)
	Rarely	3 (3.15)
B21	While breaking bad news, I tend to give information in small portions rather than doing so abruptly	
	Always	32 (33.68)
	Often	38 (40.00)
	Sometimes	14 (14.73)
	Occasionally	10 (10.52)
	Rarely	1 (1.05)
B22	After breaking bad news, I address/attend to patient's emotional reaction with full patience	
	Always	41 (43.15)
	Often	34 (35.78)
	Sometimes	13 (13.68)
	Occasionally	6 (6.31)
	Rarely	1 (1.05)
B23	After breaking bad news, I discuss the future plan of treatment with the patients and/or attendants	
	Always	37 (38.94)
	Often	42 (44.21)
	Sometimes	13 (13.68)
	Occasionally	2 (2.10)
	Rarely	1 (1.05)
B24	While communicating with nurses, paramedical staff, and other supporting staff, I display appropriate courtesy	
	Always	73 (76.84)
	Often	18 (18.94)
	Sometimes	3 (3.15)
	Occasionally	1 (1.05)
	Rarely	0

(Continued to the next page)

Supplement 2. Continued

Q. no.	Question	No. (%)
Section B		
B25	While communicating with nurses, paramedical staff, and other supporting staff, I highlight that their role and responsibility is equally important	
	Always	54 (56.84)
	Often	32 (33.68)
	Sometimes	6 (6.31)
	Occasionally	1 (1.05)
	Rarely	2 (2.10)
B26	I avoid criticising colleagues or having debates in front of patients or attendants	
	Always	67 (70.52)
	Often	20 (21.05)
	Sometimes	6 (6.31)
	Occasionally	2 (2.10)
	Rarely	0
B27	To motivate nurses, paramedical staff and other supporting staff; I teach them the principles of management of commonly encountered diseases in the ward	
	Always	40 (42.10)
	Often	31 (32.63)
	Sometimes	13 (13.68)
	Occasionally	9 (9.47)
	Rarely	2 (2.10)
B28	As a part of giving feedback, I regularly express appreciation for nurses, paramedical staff, and other supporting staff	
	Always	35 (36.84)
	Often	36 (37.89)
	Sometimes	17 (17.89)
	Occasionally	4 (4.21)
	Rarely	5 (5.26)
B29	As a part of giving feedback, I don't hesitate to give positive criticism/constructive suggestion to my subordinates and supporting healthcare staff	
	Always	37 (38.94)
	Often	36 (37.84)
	Sometimes	8 (8.42)
	Occasionally	13 (13.68)
	Rarely	1 (1.05)

Q. no.	Question	No. (%)
Section C		
C1	Lack of insight (I never felt that good communication is equally important in the management of diseases)	
	Strongly agree	24 (25.26)
	Agree	17 (17.89)
	Neutral	4 (4.21)
	Disagree	28 (29.37)
	Strongly disagree	22 (23.15)
C2	Lack of time	
	Strongly agree	49 (51.57)
	Agree	27 (28.42)
	Neutral	8 (8.42)
	Disagree	7 (7.36)
	Strongly disagree	4 (4.21)
C3	Difficulty in understanding the patient's language	
	Strongly agree	15 (15.78)
	Agree	35 (36.84)
	Neutral	16 (16.84)
	Disagree	26 (27.36)
	Strongly disagree	3 (3.15)

(Continued to the next page)

Supplement 2. Continued

Section C		
Q. no.	Question	No. (%)
C4	Human failings like stress and fatigue	
	Strongly agree	32 (33.68)
	Agree	44 (46.31)
	Neutral	14 (14.73)
	Disagree	5 (5.26)
	Strongly disagree	0
C5	Infrastructural deficits like lack of proper place for discussion, overcrowding	
	Strongly agree	52 (54.73)
	Agree	32 (33.68)
	Neutral	5 (5.26)
	Disagree	6 (6.31)
	Strongly disagree	0
C6	Long working hours	
	Strongly agree	52 (54.73)
	Agree	22 (23.15)
	Neutral	11 (11.57)
	Disagree	8 (8.42)
	Strongly disagree	1 (1.05)
C7	Lack of subject knowledge required for fully explaining the modalities of diagnosis, treatment options, or prognosis	
	Strongly agree	12 (12.63)
	Agree	22 (23.15)
	Neutral	16 (16.31)
	Disagree	31 (32.63)
	Strongly disagree	14 (14.73)
C8	Lack of training in communication skills	
	Strongly agree	25 (26.31)
	Agree	31 (32.63)
	Neutral	14 (14.73)
	Disagree	14 (14.73)
	Strongly disagree	11 (11.57)
